# The Role of Ubiquitination on Macrophages in Cardiovascular Diseases and Targeted Treatment

**DOI:** 10.3390/ijms26094260

**Published:** 2025-04-30

**Authors:** Li Wang, Yan Zhang, Jianming Yue, Ronghua Zhou

**Affiliations:** 1 Department of Anesthesiology, West China Hospital, Sichuan University, Chengdu 610041, China; wanglijyo@163.com (L.W.); zhangyan17623@163.com (Y.Z.); yuejmyycc@163.com (J.Y.); 2 The Research Units of West China (2018RU012)-Chinese Academy of Medical Sciences, West China Hospital, Sichuan University, Chengdu 610041, China

**Keywords:** cardiovascular disease, ubiquitination, macrophage, lipoprotein, inflammation

## Abstract

Cardiovascular disease (CVD) is a leading cause of morbidity and mortality worldwide, with macrophage dysfunction playing a central role in its pathogenesis. Ubiquitination, a critical post-translational modification, regulates diverse macrophage functions, including lipoprotein metabolism, inflammation, oxidative stress, mitophagy, autophagy, efferocytosis, and programmed cell death (pyroptosis, necroptosis, ferroptosis, and apoptosis). This review highlights the regulatory roles of ubiquitination in macrophage-driven CVD progression, focusing on its effects on cholesterol metabolism, inflammation, activation, polarization, and the survival of macrophages. Targeting ubiquitination pathways has therapeutic potential by enhancing macrophage autophagy, reducing inflammation, and improving plaque stability. However, challenges, such as off-target effects, ubiquitination crosstalk, and macrophage heterogeneity, must be addressed. By integrating advances in ubiquitination biology, therapeutic strategies can be developed to mitigate CVD and other macrophage-driven inflammatory diseases. This review underscores the potential of ubiquitination-targeting therapies for mitigating CVD and highlights the key areas for further investigation.

## 1. Introduction

Cardiovascular disease (CVD) is the leading cause of morbidity and mortality worldwide. The crude prevalence of CVD was 607.64 million cases in 2020, an increase of 29.01% compared with 2010. In addition, 19.05 million deaths were estimated to have been caused by CVD globally in 2020, which amounts to an increase of 18.71% from 2010 [1]. Immune cells serve as the essential pathological mechanism for the development and progression of CVD, and monocytes or macrophages play an important role in tissue homeostasis and inflammation by balancing pro- and anti-inflammatory responses [2]. The accumulating research suggests that ubiquitination plays an imperative regulatory role in macrophage function and macrophage-orchestrated inflammation. In the following sections, we first give a brief introduction to ubiquitination, followed by a description of how ubiquitination affects the prognosis of CVD by influencing macrophage function, including functions such as lipoprotein uptake and efflux, inflammation, and so on.

## 2. Ubiquitination and Macrophages

Ubiquitination is a highly conserved and versatile post-translational modification that plays a critical role in regulating protein stability, localization, and function. This process involves the conjugation of ubiquitin (Ub) to target proteins [3]. The ubiquitination reaction is a multistep enzymatic cascade mediated by three classes of enzymes: E1 (Ub-activating enzymes), E2 (Ub-conjugating enzymes), and E3 (Ub ligases) [3]. Ub is first activated by an E1 enzyme in an ATP-dependent manner, transferred to an E2 conjugating enzyme, and subsequently ligated to a lysine residue on the target protein by an E3 ligase, which facilitates the formation of an isopeptide bond between the C-terminus of the Ub and the substrate protein [4].

The ubiquitin system is characterized by its versatility, with Ub having seven lysine residues (K6, K11, K27, K29, K33, K48, and K63) and an N-terminal methionine site that can serve as sites for ubiquitination. These residues give rise to different types of ubiquitin linkages, each with distinct biological functions [5]. For instance, K48-linked polyubiquitin chains primarily target proteins for proteasomal degradation, while K63-linked polyubiquitin chains are involved in non-proteasomal pathways, such as intracellular signaling, DNA repair, and protein–protein interactions [6].

Ubiquitination is a reversible process [7], counteracted by deubiquitinating enzymes (DUBs), which remove Ub from target proteins [8]. DUBs are classified into nine superfamilies based on their sequence and domain conservation: Ub-specific proteases (USPs), ovarian tumor proteases (OTUs), Ub C-terminal hydrolases (UCHs), Machado–Joseph domain-containing proteases (MJDs), JAMM/MPN domain-associated zinc-dependent metalloproteases (JAMMs), motif interacting with Ub-containing novel DUB family (MINDYs), monocyte chemotactic protein-induced proteins (MCPIPs), permuted papain fold peptidases of dsRNA viruses and eukaryotes (PPPDEs), and zinc finger-containing Ub peptidase 1 (ZUP1) [9]. DUBs play a critical role in regulating the stability and activity of ubiquitinated proteins, as well as maintaining cellular homeostasis by preventing excessive ubiquitination.

In the context of immune and inflammatory diseases, ubiquitination serves as a key regulatory mechanism for macrophage function. By modulating the stability and activity of signaling proteins, ubiquitination controls macrophage activation, polarization, and programmed death, which are central to the development and progression of diseases, such as atherosclerosis (AS), myocardial infarction (MI), and autoimmune disorders. This overview highlights the fundamental mechanisms of ubiquitination and deubiquitination and sets the stage for the subsequent sections that explore the specific roles of ubiquitination in macrophage-driven inflammation and disease pathogenesis.

### 2.1. Foam Cell Formation and Ubiquitination

Atherosclerosis (AS) is the most common CVD in clinics [10]. During AS, monocytes are recruited and then differentiate into macrophages in response to locally produced macrophage colony-stimulating factor (M-CSF) and other cytokines within the vascular endothelium [11]. Macrophages are an important source of foam cells in AS [12]. Macrophage foam cell formation develops from the dysregulation of three interconnected biological processes: lipoprotein uptake, cholesterol esterification, and cholesterol efflux, each of which can be regulated by ubiquitination.

#### 2.1.1. Ox-LDL Uptake and Ubiquitination

During AS, macrophages specialize in taking up large amounts of oxidized low-density lipoprotein (ox-LDL) through scavenger receptors (SRs), including lectin-like ox-LDL receptor 1 (Lox-1), SR-A, and cluster of differentiation 36 (CD36) [13], ultimately giving rise to cholesterol ester-engorged macrophages [14] known as macrophage foam cells [15,16]. In vitro experiments have shown that CD36 and SR-A are responsible for the majority (75–90%) of ox-LDL uptake by macrophages [17]. Increasing evidence suggests that ubiquitination critically modulates the function of CD36 and SR-A in ox-LDL internalization by macrophages, inhibiting foam cell formation and thus suppressing AS development. For instance, in vitro studies have shown that curcumin application is beneficial for reducing the accumulation of cholesterol in macrophages and preventing the transformation of macrophage foam cells. Moreover, these studies have revealed that SR-A ubiquitination and subsequent degradation are responsible for the inhibition of SR-A-mediated ox-LDL uptake [18]. Similarly, L-theanine has been reported to inhibit ox-LDL uptake by RAW264.7 cells via promoting SR-A ubiquitination and subsequent degradation [19]. In addition, CD36 can also undergo K48-linked polyubiquitination and subsequent proteasomal degradation, thus impeding ox-LDL uptake by macrophages. This result obtained from extracellular experiments can be further enhanced by the suppression of DUBs, including USP10, USP11, USP14, and UCHL1 [20,21,22,23]. In addition to enabling the proteasomal degradation of SRs, ubiquitination also exerts a vital role in the endocytosis of various surface receptors, including SRs. In vitro data have suggested that the K63-linked polyubiquitination of SR-A1, enhanced by the genetic or pharmacological inhibition of USP9X, increases SR-A1 cell surface internalization after the binding of ox-LDL, thereby promoting foam cells formation [24].

Moreover, macrophages’ uptake of ox-LDL can also be orchestrated by the ubiquitination of the upstream signaling of SRs. For instance, in vitro data have shown that the ubiquitinating and degrading suppressor of cytokine signaling 1/3 (SOCS1/3) induced by tripartite motif-containing protein (TRIM) 13 is able to mediate CD36 expression, increasing intracellular cholesterol accumulation [25]. Additionally, in vitro data have shown that intermedin can suppress phosphatase and tensin homolog (PTEN) ubiquitination and degradation, thereby decreasing SR-A expression and acetylated LDL (ac-LDL) uptake [26].

**Figure 1:**
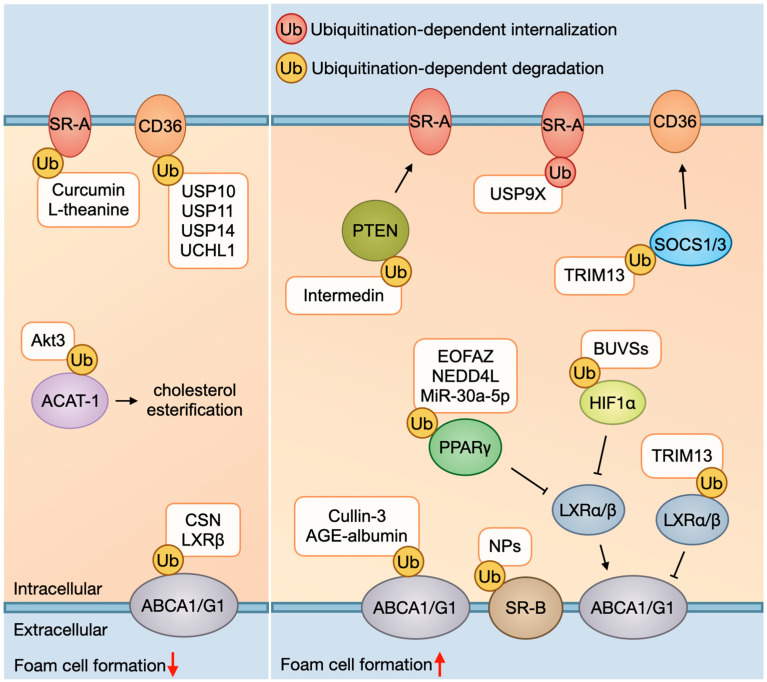
Foam cell formation and ubiquitination. As for ox-LDL uptake, ubiquitination-dependent SRs (e.g., SR-A and CD36) degradation mediated by curcumin, L-theanine, and DUBs (e.g., USP10, USP11, USP14, and UCHL1) usually decreases ox-LDL uptake, whereas ubiquitination-dependent SRs internalization mediated by USP9X increases it. In addition, PTEN and SOC1/3 are negative regulators of SRs; ubiquitination and subsequent degradation of them mediated by intermedin and TRIM13 can promote SRs to uptake ox-LDL. As for cholesterol efflux, ubiquitination-dependent transporters’ (ABCA1, ABCG1, and SR-B) degradation mediated by cullin-3, AGE-albumin, and NPs exposure usually decreases cholesterol outflow. In addition, ABCA1/G1 is target gene of LXR (liver X receptor) α/β and can be modulated by LXRα/β. LXRβ can inhibit ubiquitination and degradation of ABCA1, while ubiquitinating and degrading of LXRα/β mediated by TRIM13 can inhibit ABCA1/G1. PPARγ and HIF1α are upstream signaling of LXRα/β; ubiquitination and degradation of them mediated by EOFAZ, NEDD4L, MiR-30a-5p, and BUVSs can inhibit LXRα/β and then suppress ABCA1/G1. Abbreviations: ox-LDL, oxidized low-density lipoprotein; SR, scavenger receptor; CD36, cluster of differentiation 36; PTEN, phosphatase and tensin homolog; SOCS1/3, suppressor of cytokine signaling 1/3; ABC, ATP-binding cassette transporters; LXR, liver X receptor; PPARγ, peroxisome proliferator-activated receptor γ; HIF1α, hypoxia-inducible factor 1α; USP, ubiquitin-specific proteases; UCH, ubiquitin C-terminal hydrolases; AIBP, apolipoprotein A-1 binding protein; CSN, COP9 signalosome; EOFAZ, essential oil from Fructus Alpinia zerumbet; TRIM13, tripartite motif-containing protein 13; BUVS, benzotriazole ultraviolet stabilizer; AGE, advanced glycation end product.

In summary, the process of ox-LDL uptake can be influenced by the ubiquitination of SRs and their upstream signaling molecules in macrophages. Ubiquitination-mediated SRs degradation usually decreases ox-LDL uptake and foam cells formation, thereby retarding AS development, whereas ubiquitination-mediated SRs internalization increases it. Furthermore, the expression of SRs can be modulated by the ubiquitination of their upstream signals, such as SOCS1/3 and PTEN, which has only been investigated in a few articles and thus deserves further exploration (Figure 1).

#### 2.1.2. Cholesterol Esterification and Ubiquitination

During the formation of foam cells, dysfunctions in cholesterol synthesis and cholesterol ester hydrolysis also contribute to excessive intracellular cholesterol accumulation in macrophages. Acyl-coenzyme A: cholesterol acyltransferase-1 (ACAT-1) is an enzyme that diverts cholesterol from the cholesterol efflux pathway by esterifying it and promoting its storage in the endoplasmic reticulum [27]. In vitro experiments by Ding et al. have revealed that Akt3 can restrict cholesterol esterification in macrophages and suppress foam cell formation by promoting ACAT-1 degradation via ubiquitination [28]. However, limited research has been conducted on the role of ubiquitination in regulating the cholesterol esterification process, indicating a potential area for further investigation.

#### 2.1.3. Cholesterol Efflux and Ubiquitination

In addition to taking up ox-LDL, macrophage foam cells can efflux intracellular cholesterol via a range of transporters, including ATP-binding cassette transporters (ABC) A1, ABCG1, and SR-B in mammalian cells [29]. Effective cholesterol export is crucial for maintaining cellular cholesterol homeostasis since the majority of cell types are incapable of metabolizing cholesterol. This process is vital for reducing intracellular cholesterol levels and preventing cholesterol accumulation within cells [30]. Impaired cholesterol outflow due to a malfunction of these transporters in macrophages leads to the conversion of macrophages into foam cells [29], and ubiquitination plays an important role in regulating the function of these transporters [31].

On the one hand, the ubiquitination of the cholesterol transporters themselves regulates their functions. The ubiquitination and subsequent proteasomal degradation of ABCA1/G1 are decreased by cell cholesterol loading, thus increasing cellular cholesterol export and inhibiting the accumulation of intracellular cholesterol [32]. In vitro data have indicated that there remains an acute fine-tuning of cholesterol transporter activity in cholesterol-loaded cells in response to fluctuations in intracellular cholesterol levels [32]. However, in vitro studies have demonstrated that under conditions of high cellular cholesterol content, ABCA1 becomes susceptible to ubiquitination and lysosomal degradation, thereby inhibiting cholesterol outflow [33]. In addition, experiments both in vivo and in vitro conducted by Raghavan et al. have shown that thrombin–protease-activated receptor 1 (Par1) signaling contributes to the ubiquitination and degradation of ABCA1 via E3 Ub ligase cullin 3, reducing cholesterol outflow [34]. Furthermore, in vitro data have suggested that COP9 signalosome (CSN) inhibits the ubiquitination and degradation of ABCA1, whereas the advanced glycation end product (AGE) albumin facilitates it [35,36]. In addition, exposure to magnetite nanoparticles (NPs) has been shown to elicit the ubiquitination and subsequent degradation of SR-B1, leading to a reduction in cholesterol efflux and abnormal cholesterol accumulation in RAW264.7 cells and THP-1 cells [37].

On the other hand, the function of cholesterol efflux transporters can also be regulated by the ubiquitination of their upstream signaling. Hypoxia-inducible factor 1α (HIF1α), for instance, undergoes ubiquitination and degradation in the presence of benzotriazole ultraviolet stabilizers (BUVSs) in RAW264.7 cells, thus downregulating the expression of LXRα/β and their target genes ABCA1/G1. This results in diminished cholesterol efflux and increased foam cell formation [38]. Likewise, the ubiquitination and degradation of LXRα/β itself, mediated by the E3 Ub ligase TRIM13, plays a pivotal role in cholesterol homeostasis regulation. Specifically, TRIM13, via the ubiquitination and degradation of LXRα/β, represses the transcription of ABCA1/G1 and thereby impedes cholesterol efflux in vitro [25]. Additionally, ubiquitination-mediated PPARγ destabilization negatively impacts the PPARγ-LXRα-ABCA1/G1 pathway. In vitro treatment with essential oil from Fructus Alpinia zerumbet (EOFAZ) has been observed to reduce PPARγ ubiquitination and degradation, enhancing its stability and reactivating the cholesterol efflux machinery [39]. Similarly, PPARγ ubiquitination has been reported to decrease the expression of ABCA1 and ABCG1, leading to foam cell formation. In vitro data have shown that MiR-30a-5p treatment can repress PPARγ ubiquitination by E3 Ub ligase NEDD4L repression, upregulating the expression of ABCA1, ABCG1, LDLR, and PCSK9, and attenuating lipid accumulation in macrophages [40].

In conclusion, ubiquitination serves as a master regulator of macrophages, influencing cholesterol metabolism and atherogenesis. Promoting the ubiquitination-dependent degradation of ox-LDL uptake receptors, inhibiting ubiquitination-dependent cholesterol esterification, or inhibiting the ubiquitination-dependent degradation of cholesterol efflux transporters can be effective for inhibiting foam cell formation and thereby retarding AS development, and the search for such a drug or target has great research potential.

### 2.2. Inflammation and Ubiquitination

Macrophage inflammation has been studied extensively in both in vitro and in vivo models of CVD, leading to the simplified notion that M1 macrophages promote inflammation, while M2 macrophages contribute to its resolution [41]. There is growing evidence that highlights how macrophage inflammation and CVD prognosis are tightly regulated by ubiquitination, which can modulate protein stability, activity, and signaling pathways.

#### 2.2.1. cGAS-STING Pathway and Ubiquitination

**Figure 2:**
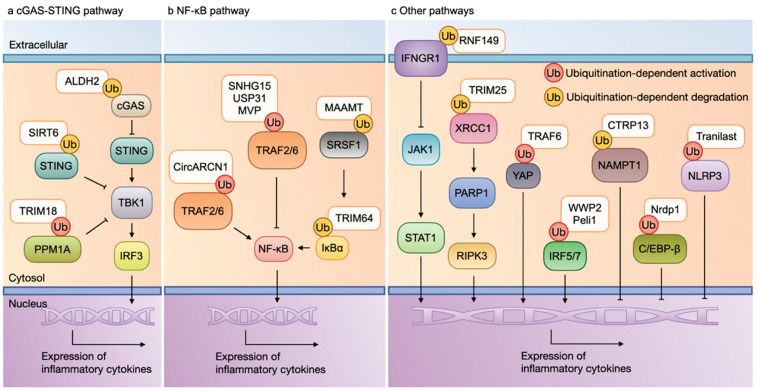
Macrophage inflammation and ubiquitination. (**a**) cGAS-STING pathway and ubiquitination: The activation of the cGAS-STING pathway contributes to the activation of the TBK1-IRF3 pathway, thereby resulting in the production of inflammatory cytokines. The ubiquitination of cCAS, PPM1A, and STING mediated by ALDH2, SIRT6, and TRIM18 can suppress the expression of inflammatory cytokines. (**b**) NF-κB pathway and ubiquitination: The activation of TRAF2/6 contributes to the activation of MAPK and NF-κB, leading to the expression of inflammatory molecules. The ubiquitination of either TRAF2/6, SRSF1, or IκBα mediated by CircARCN1, SNHG15, USP31, MVP, MAAMT, and TRIM64 can stimulate the pro-inflammatory phenotype of a macrophage. (**c**) Other pathways and ubiquitination: The ubiquitination of IFNGR1, XRCC1, IRF5/7, and YAP mediated by RNF149, TRIM25, TRAF6, WWP2, and Peli1 promote, while the ubiquitination of NAMPT1, C/EBP-β, and NLRP3 mediated by CTRP13, Nrdp1, and tranilast inhibit, the expression of inflammatory cytokines. Abbreviations: cGAS, cyclic GMP-AMP synthase; STING, stimulator of interferon genes; TBK1, TANK-binding kinase 1; IRF3, interferon regulatory factor 3; PPM1A, protein phosphatase 1A; NF-κB, nuclear factor-κB; TRAF, tumor necrosis factor receptor-associated factor; IκB, inhibitor of κB; MAPK, mitogen-activated protein kinase; SRSF1, serine/arginine-rich splicing factor 1; IFNGR1, interferon gamma receptor 1; YAP, Yes-associated protein; C/EBP-β, CCAAT enhancer-binding protein-β; NLRP3, NOD-like receptor thermal protein domain-associated protein 3; MVP, major vault protein; TRIM, tripartite motif-containing protein; USP, ubiquitin-specific proteases; RNF149, ring finger protein 149; CTRP13, C1q/TNF-related protein 13; Nrdp1, neuregulin receptor degradation protein 1.

The activation of the cyclic GMP-AMP synthase (cGAS)-stimulator of interferon genes (STING) pathway contributes to activation of the TANK-binding kinase 1 (TBK1)–interferon regulatory factor 3 (IRF3) pathway, thereby resulting in the production of inflammatory cytokines [42]. Numerous studies have been conducted to explore the effects of ubiquitination on this pathway, as summarized in Figure 2a.

In vitro, the level of K48-linked polyubiquitination and the degradation of cGAS are decreased, leading to the enhanced stability of cGAS and the activation of the cGAS-STING pathway. However, ALDH2 stimulation reduces the interaction between USP14 and cGAS, thereby accelerating the polyubiquitination of cGAS and promoting its degradation. This process suppresses the activation of the cGAS-STING pathway and dephosphorylates and inactivates the TBK1-IRF3 pathway, ultimately attenuating pro-inflammatory cytokine production in macrophages [43]. Consistent with these findings, enhanced cGAS stability and increased atherosclerotic plaque formation have been observed in vivo, highlighting the therapeutic potential of targeting cGAS ubiquitination in CVD [43]. In addition, the ubiquitination and degradation of STING have been reported to influence the prognosis of myocardial infarction (MI). In vitro studies have demonstrated that SIRT6 deacetylates the STING protein, resulting in decreased ubiquitination and a stabilized STING protein, while in a murine model of MI, STING inhibition has led to alleviated cardiac dysfunction and adverse remodeling by inhibiting the release of pro-inflammatory cytokines post-MI [44]. These findings suggest that targeting STING ubiquitination may serve as a promising strategy for managing post-MI cardiac injury.

Ubiquitination also has implications for CVD by affecting the interaction of STING with its downstream signaling pathways. For instance, the E3 Ub ligase TRIM18 has been shown to recruit and stabilize protein phosphatase 1A (PPM1A) by mediating K63-linked ubiquitination. This interaction leads to the dephosphorylation and inactivation of TBK1, blocking its interaction with upstream adaptors, such as mitochondrial antiviral signaling (MAVS) and STING, and dampening type-I interferon (IFN) production via dephosphorylating IRF3. In in vitro experiments, TRIM18 overexpression has been reported to strongly enhance PPM1A ubiquitination and reduce type-I IFN responses in response to viral infection, while in in vivo studies, TRIM18-deficient mice have exhibited enhanced antiviral immunity and reduced susceptibility to viral myocarditis [45]. These findings highlight the role of ubiquitination in regulating innate immune responses and their implications for CVD.

The interferon regulatory factor (IRF) family is one of the downstream signals of the cGAS-STING pathway and can also be modified by ubiquitination, thus playing an important role in regulating macrophage inflammation responses. For instance, WWP2 is an E3 Ub ligase and can interact with IRF7, promoting its non-degradative ubiquitination, nuclear translocation, and transcriptional activity. In in vitro studies, this process has been shown to upregulate CCL5 and IFN signaling, leading to increased infiltration, pro-inflammatory activation, and the profibrotic potential of cardiac macrophages. In in vivo studies using hypertension-induced non-ischemic cardiomyopathy (NICM) mouse models, WWP2 deficiency has reduced cardiac fibrosis and ameliorated disease progression, suggesting that WWP2-mediated IRF7 ubiquitination is a key driver of fibrosis in NICM [46]. Moreover, in macrophages, Peli1 promotes M1 polarization and migratory ability by enhancing K63-linked ubiquitination and the nuclear translocation of IRF5. Conversely, in vivo, Peli1 deletion in macrophages protects against myocardial ischemia/reperfusion (I/R) injury by reducing IRF5-mediated inflammation and macrophage infiltration [47]. These findings provide mechanistic insights into the role of IRF5 ubiquitination in macrophage polarization and its impact on myocardial I/R injury. Additionally, numerous studies have investigated the role of other members of the IRF family in macrophages in the pathology of murine CVD. For example, IRF5 promotes the maintenance of pro-inflammatory CD11c+ macrophages within atherosclerotic lesions [48], contributing to the formation of rupture-prone plaques in AS [49]. In contrast, a deficiency of IRF3 and IRF1 in macrophages ameliorates AS by inhibiting the pro-inflammatory properties of macrophages [50,51]. Furthermore, IRF9 inhibition in macrophages induces M2-like polarization, enhancing angiogenesis, arteriogenesis, and perfusion recovery in experimental peripheral artery disease (PAD) [52]. In microglia, the downregulation of IRF5 and the upregulation of IRF4 promote M2 activation, quenching pro-inflammatory responses and improving outcomes after ischemic stroke [53]. And so the ubiquitination of these IRF family members may be a detailed mechanism by which to modulate the prognosis of CVD. These studies collectively highlight the intricate role of IRF family ubiquitination in modulating macrophage function and CVD prognosis.

#### 2.2.2. NF-κB Pathway and Ubiquitination

Upon IL-1 stimulation, the IL-1 receptor (IL-1R) recruits multiple adaptor molecules into signaling complexes and activates tumor necrosis factor receptor-associated factor (TRAF) 2/6, which are pivotal signaling proteins in the activation of mitogen-activated protein kinase (MAPK) and nuclear factor-κB (NF-κB) [54]. In the canonical pathway, NF-κB is bound and inhibited by the inhibitory inhibitor of κB (IκB) proteins in the cytoplasm. Upon activation, IκB is degraded, freeing the active NF-κB to translocate to the nucleus and induce the expression of the target genes [55]. A large number of studies have explored the effect of ubiquitination on this pathway and its subsequent influence on inflammatory cytokine production (Figure 2b).

IκB can be degraded via ubiquitination, thus affecting the macrophage inflammatory response. For instance, in vitro studies have demonstrated that TRIM64 directly interacts with IκBα and promotes IκBα ubiquitination, which activates NF-κB signaling and promotes macrophage inflammation [56].

In addition, the upstream signaling of NF-κB can be modified by ubiquitination and then affects the prognosis of CVD. For instance, in vitro data have shown that MAAMT knockdown inhibits the ubiquitination-mediated degradation of serine/arginine-rich splicing factor 1 (SRSF1), increasing its protein expression. This action restrains the activation of the NF-κB pathway, thereby inhibiting the pro-inflammatory activation of macrophages. In mice with experimental autoimmune myocarditis (EAM), MAAMT knockdown has reduced macrophage recruitment and pro-inflammatory activation, ameliorated reversed ventricular remodeling, and improved cardiac function [57]. These studies collectively highlight the intricate role of NF-κB axis ubiquitination in modulating macrophage function and CVD prognosis.

Moreover, many studies have focused on the effects of TRAF2/6 ubiquitination on macrophage function. For example, in vitro studies have demonstrated that the small nucleolar RNA host gene 15 (SNHG15) interacts with TRAF2 to hide its ubiquitination regions, thereby decreasing the K63-linked ubiquitination of TRAF2. This interaction represses the activation of the MAPK and NF-κB signaling pathways, attenuating inflammatory responses and promoting M2 macrophage polarization. In contrast, silencing SNHG15 in a murine model of acute ischemic stroke has accelerated macrophage polarization toward the M1 phenotype, improving stroke-induced immunosuppression and decreasing susceptibility to stroke-associated infections [58]. Additionally, in vitro studies have shown that macrophage-expressed circARCN1 affects the interaction between HuR and USP31 mRNA, downregulating the deubiquitinating enzyme USP31. This process induces the K63-linked ubiquitination of TRAF2/6 and NF-κB activation, promoting macrophage inflammatory responses. Meanwhile, the macrophage deletion of circARCN1 in a murine model of AS has markedly decreased macrophage accumulation and inflammation, thus ameliorating atherosclerotic lesions [59]. Conversely, in vitro data have revealed that the major vault protein (MVP) interacts with TRAF6 and prevents the K63-linked ubiquitination of TRAF6. This process suppresses the activity of TRAF6 and the inflammatory responses of macrophages, while a global or myeloid-specific MVP gene knockout has aggravated AS in mice, accompanied with increased macrophage infiltration and heightened inflammatory responses [60]. These findings highlight the regulatory role of the TRAF family of proteins and their ubiquitination for modulating macrophage-driven inflammation.

In addition to being capable of being modified by ubiquitination itself, the TRAF family also binds to and ubiquitinates Yes-associated proteins (YAPs). In vitro experiments have shown that as an E3 Ub ligase, TRAF6 can interact with YAPs and induce the K63-linked ubiquitination of YAPs, increasing YAPs nuclear localization and protein stability. This process upregulates chemokine (e.g., CCL2, CCL7, CXCL1, CXCL3, CXCL5, and CXCL12) production and monocyte/macrophage migration. Moreover, YAPs overexpression in myeloid cells increases the atherosclerotic lesion size and infiltration of macrophages, whereas a YAPs deficiency abrogates atherosclerotic plaque in AS models [61]. These studies highlight the intricate role of YAPs ubiquitination in modulating macrophage function and CVD prognosis. Notably, there are still many ongoing studies addressing the close relationship between YAPs activation and CVD. It has been reported that YAPs activation enhances the pro-inflammatory response and impairs the reparative response of macrophages, which in turn augments cardiac fibrosis and adverse remodeling following an MI [62]. While YAPs suppression or knockdown shifts polarization toward a resolving phenotype and disrupts inflammasome induction in macrophages, which in turn reduces the incidence of aortic dissection, it improves the systolic function pathological remodeling in NICM [63], and attenuates MI-induced injury [64]. These findings suggest that YAPs ubiquitination may play a critical role in regulating macrophage activation and inflammation in cardiovascular diseases, warranting further investigation.

#### 2.2.3. Other Pathways and Ubiquitination

Interferon gamma receptor 1 (IFNGR1) serves as a crucial receptor protein in the type-II IFN signaling pathway. Upon stimulation by IFN-γ, IFNGR1 activates Janus kinase 1 (JAK1), leading to signal transducer and activator of transcription 1 (STAT1) phosphorylation and the subsequent induction of numerous pro-inflammatory genes. IFNGR1 is also one of the ubiquitination-modified proteins. Ring finger protein 149 (RNF149), an E3 Ub ligase, induces K48-linked polyubiquitination and the subsequent proteasomal degradation of IFNGR1, which contributes to restrain the inflammatory responses in macrophages. However, an RNF149 loss-of-function was shown to aggravate myocardial dysfunction and adverse remodeling via enhancing pro-inflammatory macrophage activation in a murine model of myocardial I/R injury [65]. These findings suggest that the RNF149-mediated ubiquitination of IFNGR1 plays a protective role in post-MI cardiac recovery by attenuating excessive inflammation (Figure 2c).

XRCC1, an important DNA repair enzyme, plays a vital role in inhibiting the activation of the necroptosis pathway through suppressing the toxic effect of PARP1. XRCC1 can also be modified by ubiquitination. In vitro studies have shown that TRIM25 promotes XRCC1 ubiquitination and degradation, mediating M1 polarization and the necroptosis of macrophages. However, TRIM25 deficiency has been reported to alleviate atherosclerotic plaque in mice with AS through inhibiting the programmed death and pro-inflammatory activation of macrophages [66]. These findings highlight the role of XRCC1 ubiquitination in driving macrophage-mediated inflammation and atherosclerosis.

The CCAAT enhancer-binding protein-b (C/EBP-β), an important transcriptional regulatory element of Arg1, also undergoes ubiquitination modification. Neuregulin receptor degradation protein 1 (Nrdp1), an E3 Ub ligase, effectively promotes C/EBP-β ubiquitination, mediating Arg1 expression and M2 polarization. This action improves brain edema and promotes the recovery of impaired neurological function after an intracerebral hemorrhage (ICH) [67]. These findings suggest that the Nrdp1-mediated ubiquitination of C/EBP-β has a protective role in mitigating neuroinflammation and improving outcomes after an ICH.

Nicotinamide phosphoribosyl-transferase 1 (NAMPT1) is a rate-limiting enzyme that converts nicotinamide to nicotinamide mononucleotide in all organisms [68]. C1q/tumor necrosis factor (TNF)-related protein-13 (CTRP13) has been reported to upregulate the expression and activity of NAMPT1 through inhibiting its ubiquitination and degradation in macrophages. Moreover, in rodent abdominal aortic aneurysm (AAA) models, CTRP13 has reduced the incidence and severity of AAAs in conjunction with attenuated aortic macrophage infiltration and the expression of pro-inflammatory cytokines [69]. These findings indicate that the CTRP13-mediated regulation of NAMPT1 ubiquitination has a protective role in AAA development.

The aberrant activation of the nucleotide-binding oligomerization domain, leucine-rich repeat-containing receptor family pyrin domain-containing 3 (NLRP3) inflammasome, is thought to play a pathogenic role in AS [70]. In vitro studies have shown that tranilast treatment increases NLRP3 ubiquitination, inhibits the assembly and activation of the NLRP3 inflammasome, and ameliorates macrophage inflammation. Moreover, mice with AS receiving tranilast treatment have displayed a significant reduction in their atherosclerotic lesion size, in conjunction with a decreased expression and activation of the NLRP3 inflammasome and expression of inflammatory molecules [71]. These findings highlight the therapeutic potential of targeting NLRP3 ubiquitination to mitigate inflammation and AS progression.

Collectively, these studies highlight the intricate role of ubiquitination in modulating macrophage-driven inflammation and its impact on cardiovascular disease (CVD) and other inflammation-related conditions. Given the central role of macrophages in orchestrating inflammation, targeting the ubiquitination pathways may offer novel therapeutic strategies for managing CVD and other inflammatory disorders.

### 2.3. Oxidative Stress and Ubiquitination

Oxidative stress, characterized by the overproduction of reactive oxygen species (ROS), promotes the oxidation of lipids, proteins, and DNA, leading to cellular damage and dysfunction. It has been implicated in the development and progression of CVD [72]. Nuclear factor erythroid 2-related factor 2 (Nrf2) is a key transcription factor that regulates cellular redox homeostasis and exhibits potent anti-oxidative and anti-inflammatory activities. Under normal physiological conditions, Nrf2 interacts with Kelch-like ECH-associated protein 1 (Keap1) in the cytoplasm, forming a low-activity complex [73]. However, upon oxidative stress, Keap1 undergoes degradation, leading to the dissociation of Nrf2 from the Nrf2/Keap1 complex and its translocation to the nucleus. In the nucleus, Nrf2 induces the expression of numerous anti-oxidative and anti-inflammatory genes, mitigating oxidative damage [74]. The pharmacological modulation of the Nrf2/Keap1 pathway has been shown to influence Nrf2 ubiquitination and stability. For instance, oridonin treatment has been reported to increase the stability of Nrf2 by blocking Nrf2 ubiquitination, reducing oxidative stress in macrophages and, thus, retarding AS [75]. These findings highlight that the Nrf2/Keap1 pathway is a key regulator of oxidative stress and that regulation of the ubiquitination of the Nrf2/Keap1 pathway may also be a potential target for CVD treatment.

### 2.4. Programmed Cell Death and Ubiquitination

#### 2.4.1. Apoptosis and Ubiquitination

Macrophage foam cells frequently undergo apoptosis or necrosis, contributing to the formation of a growing “necrotic core” composed of cholesterol esters, cholesterol crystals, and cell debris. This necrotic core promotes plaque initiation, growth, and instability, leading to severe complications, such as angina, myocardial infarction (MI), and stroke [10]. The regulation of macrophage cell death pathways is critical for AS progression, and ubiquitination plays a pivotal role in modulating these processes. For instance, the aggregated LDL (ag-LDL) was reported to facilitate foam cell formation and AS by restraining the apoptosis of lipid-bearing macrophages [76]. However, this process can be reversed by LDL-inducible gene (LIG) suppression, which inhibits the ubiquitination and degradation of p53, a pro-apoptotic transcription factor. In vitro studies have shown that LIG contributes to foam cell formation by the suppression of the apoptosis of lipid-bearing macrophages via p53 ubiquitination and degradation [77]. Moreover, neointimal hyperplasia, characterized by a thickening of the arterial wall and a reduction in the arterial lumen space, is the main cause of restenosis after a percutaneous coronary intervention (PCI) [78]. And IFN-γ has been reported to aggravate neointimal hyperplasia. In vitro data have shown that IFN-γ significantly increases the degradation and polyubiquitination of liver X receptor (LXR) α, inducing endoplasmic reticulum (ER) stress-mediated apoptosis in macrophages. Conversely, IFN-γ suppression ameliorates neointimal hyperplasia by inhibiting macrophage infiltration and cellular apoptosis in mice with experimental arterial restenosis [79]. These findings highlight the therapeutic potential of targeting the ubiquitination pathways to regulate macrophage apoptosis and CVD, offering new strategies to mitigate CVD progression.

#### 2.4.2. Necroptosis and Ubiquitination

Macrophages in atherosclerotic plaques are prone to necroptosis, a form of programmed cell death that promotes the release of pro-inflammatory cytokines and chemokines, further exacerbating inflammation and AS progression. Targeting the necroptosis pathway by ubiquitination delays the progression of AS. As mentioned before, in addition to mediating M1 polarization, XRCC1 ubiquitination and degradation induced by TRIM25 can also mediate the necroptosis of macrophages. And the inhibition of necroptosis is involved in attenuating atherosclerotic plaques in AS mice [66]. These findings highlight the therapeutic potential of targeting the necroptosis pathways to mitigate inflammation and AS progression.

#### 2.4.3. Ferroptosis and Ubiquitination

Macrophages in atherosclerotic plaques are also prone to ferroptosis, a form of iron-dependent cell death driven by oxidative stress and lipid peroxidation. Ferroptosis promotes the release of pro-inflammatory cytokines and chemokines, further exacerbating inflammation and AS progression [80]. The Nrf2/Keap1 signaling pathway plays a critical role in protecting against ferroptosis by regulating lipid peroxidation and iron metabolism [81]. In vitro data have shown that Panax notoginseng saponins (PNS) treatment suppresses the ubiquitination and degradation of Keap1 by inhibiting the deubiquitinase USP2, leading to Nrf2 activation. This process enhances resistance to ferroptosis and ferroptosis-aggravated foam cell formation inflammation. Moreover, in vivo studies have revealed that PNS inhibits ferroptosis and atherosclerosis [82]. These findings highlight the therapeutic potential of targeting Keap1 ubiquitination to mitigate ferroptosis and its contribution to AS.

#### 2.4.4. Pyroptosis and Ubiquitination

Pyroptosis, a form of inflammasome-mediated programmed cell death, is closely associated with AS and contributes to necrotic core formation and plaque instability [83]. As mentioned before, in addition to enhancing macrophage inflammation, TRIM64-mediated NF-κB activation via promoting IκBα K67-linked ubiquitination induces NLR family pyrin domain-containing (NLRP) 3 activation and NLRP3-mediated macrophage pyroptosis. These findings highlight the role of TRIM64-mediated ubiquitination in regulating pyroptosis and its contribution to AS.

### 2.5. Autophagy and Ubiquitination

Autophagy dysfunction in AS models has been associated with increased lipid accumulation, apoptosis, and inflammation [84]. The stability of sirtuin 1 (Sirt1), a key regulator of autophagy, is maintained by the C1q/TNF-related protein 9 (CTRP9) via upregulation of the deubiquitinase USP22. In vitro studies have shown that CTRP9-activated USP22 inhibits the ubiquitination and degradation of Sirt1, thereby enhancing macrophage autophagy. This process attenuates the ox-LDL-induced impairment of cell viability, inhibition of autophagy, and increased lipid accumulation [85]. These findings suggest that targeting Sirt1 ubiquitination through CTRP9/USP22 may provide a novel strategy for promoting macrophage autophagy and reducing AS severity.

### 2.6. Mitophagy and Ubiquitination

Mitophagy, a cellular process that eliminates damaged mitochondria through autophagy, is crucial for maintaining mitochondrial quality and preventing disease development [86]. Mitofusin (MFN) 1/2 are key components in the process of mitophagy and are substrates that can be modified by ubiquitination. In vitro data have shown that the APOA-I binding protein (AIBP) promotes the interaction between the E3 Ub ligase parkin 2 (PARK2) and MFN1/2. This interaction facilitates the ubiquitination and degradation of MFN1/2, promoting the formation of autophagosomes to remove dysfunctional mitochondria. By reducing mitochondrial ROS and improving mitochondrial function, this process contributes to the alleviation of AS [87]. These studies highlight the critical role of ubiquitination in regulating MFN1/2-mediated mitophagy and its impact on mitochondrial health and disease progression.

### 2.7. Efferocytosis and Ubiquitination

The efficient clearance of apoptotic cells within atherosclerotic lesions is crucial for maintaining a healthy tissue microenvironment and preventing disease progression. Efferocytosis, the process by which phagocytic cells engulf and clear apoptotic cells, plays a critical role in limiting inflammation and promoting tissue repair. When apoptotic cells are not promptly cleared through efficient efferocytosis, they undergo secondary necrosis, releasing pro-inflammatory mediators and contributing to the progression of AS [88]. Enhancing efferocytosis has been shown to inhibit foam cell accumulation, reduce the release of pro-inflammatory cytokines, and limit AS progression. And ubiquitination is involved in modulating AS development by manipulating efferocytosis.

For instance, low-density lipoprotein receptor-related protein 1 (LRP-1) is a key efferocytosis receptor that facilitates the recognition and engulfment of apoptotic cells. However, LRP-1 can be ubiquitinated by epsins, which targets it for internalization and proteasomal degradation. In vitro studies have shown that ox-LDL treatment increases LRP-1 ubiquitination, which interacts with epsin and undergoes ubiquitin-dependent internalization and the downregulation of LRP-1. This action decreases efferocytosis and the efferocytosis-mediated anti-inflammatory macrophage phenotype. However, the myeloid-specific deletion of epsins enhances atheroma stability and ameliorates AS development in mice [89]. These findings highlight that modulating LRP-1 ubiquitination in macrophages is an important step in developing new therapies for AS treatment.

In addition to LRP-1, the peroxisome proliferator-activated receptor γ (PPARγ), a transcription factor known to promote anti-inflammatory and efferocytosis-related gene expression, is also regulated by ubiquitination. In vitro data have demonstrated that the deletion of Src homology 2-containing protein tyrosine phosphatase 2 (SHP2) increases the ubiquitination and degradation of PPARγ, enhancing pro-inflammatory activation and impairing the efferocytosis of macrophages. Similarly, in in vivo experiments, the deletion of macrophage-specific SHP2 has aggravated AS, with increased plaque macrophages and apoptotic cells [90]. These results indicate that efferocytosis can be influenced by ubiquitination and thus can affect cell function and disease prognosis.

## 3. Conclusions and Perspectives

Ubiquitination is a highly conserved and versatile post-translational modification that plays a critical role in regulating macrophage function and its impact on the progression and prognosis of CVD (Table 1). By modulating ubiquitination pathways, it is possible to influence macrophage activation, polarization, and inflammatory responses, thereby mitigating CVD progression. For instance, targeting the ubiquitination of key signaling molecules, such as Nrf2, PARK2, or Keap1, can enhance the anti-inflammatory and anti-oxidant responses of macrophages, reducing plaque instability and inflammation in atherosclerosis. Similarly, regulating the ubiquitination pathways that control programmed cell death, such as pyroptosis, necroptosis, and ferroptosis, offers novel therapeutic opportunities to limit tissue damage and improve repair mechanisms in CVD. These findings highlight the potential of ubiquitination-targeting therapies to address key pathophysiological processes in CVD.

However, translating these experimental insights into clinical applications is not without challenges. First, the development of ubiquitin-related inhibitors or modulators must carefully account for potential off-target effects, as ubiquitination is involved in a wide range of cellular processes, and a broad-spectrum inhibition could lead to unintended consequences. For example, targeting deubiquitinating enzymes (DUBs), such as USP22, may enhance the stability of Sirt1 and promote macrophage autophagy, but it could also interfere with other critical cellular functions regulated by USP22. Second, the specificity of targeting specific E3 ubiquitin ligases or DUBs in macrophages remains a significant hurdle, given the shared expression of many ubiquitination-related genes across different tissues. This lack of tissue specificity may lead to the non-selective modulation of ubiquitination pathways, potentially affecting normal cellular functions in other tissues.

Another critical consideration is the complex interplay between ubiquitination and other post-translational modifications, such as phosphorylation and SUMOylation, which add layers of regulatory complexity to macrophage function. For instance, phosphorylation events can modulate the activity of E3 ligases and DUBs, while SUMOylation has been shown to interact with ubiquitination pathways for regulating macrophage-driven inflammation. These crosstalk mechanisms suggest that targeting one pathway without considering its relationship with others may lead to suboptimal or even adverse therapeutic outcomes. Furthermore, the heterogeneity of macrophage populations in different disease contexts poses another challenge. Macrophages exhibit significant plasticity and functional diversity depending on their microenvironment, including polarized states, such as M1 (pro-inflammatory) and M2 (anti-inflammatory). Developing strategies to target ubiquitination pathways in a cell-type- and microenvironment-specific manner is essential to maximize therapeutic efficacy while minimizing the off-target effects.

**Table 1:** Ubiquitination and targeted treatments of macrophages in CVD in preclinical trials.

Substrate	Ubiquitin Chain Type	Detection Methods	Effects of Ubiquitination on Substrate	Effects of Ubiquitination on Macrophage	Models for Detecting Ubiquitination	Effects of Ubiquitination on Disease	Treatment Targeting Ubiquitination	Ref.
SR-A	not applicable	co-IP	promote degradation	inhibit SR-A-mediated ox-LDL uptake	J774.A1 cells	mitigate AS	curcumin	[18]
SR-A	not applicable	co-IP	promote degradation	inhibit SR-A-mediated ox-LDL uptake	RAW264.7 cells	mitigate AS	L-theanine	[19]
SR-A1	Lys63, polyUb	co-IP	promote internalization	promote SR-A1-mediated ox-LDL uptake	HEK293 cells; HeLa cell; RAW264.7 cells	aggravate AS	USP9X	[24]
CD36	polyUb	co-IP	promote degradation	inhibit CD36-mediated ox-LDL uptake	primary human macrophages	mitigate AS	CTRP9, deficiency of USP11	[20]
CD36	Lys48, polyUb	co-IP	promote degradation	inhibit CD36-mediated ox-LDL uptake	RAW264.7 cells; mouse peritoneal macrophages; THP1 macrophages; HEK293T cells	mitigate AS	UCHL1 deficiency	[21]
CD36	polyUb	co-IP	promote degradation	inhibit CD36-mediated ox-LDL uptake	RAW264.7 cells; THP1 macrophages	mitigate AS	USP14 deficiency	[22]
CD36	Lys48, polyUb	co-IP	promote degradation	inhibit CD36-mediated ox-LDL uptake	AW264.7 cells; THP1 macrophages	mitigate AS	USP10 deficiency	[23]
SOCS1/3	not applicable	co-IP	promote degradation	promote CD36 expression via STAT1 activation	mouse peritoneal macrophages	aggravate AS	TRIM13 deficiency	[25]
PTEN	polyUb	co-IP	promote degradation	promote SR-A expression and ac-LDL uptake	RAW264.7 cells; mouse peritoneal macrophages	aggravate AS	Intermedin	[26]
ACAT-1	polyUb	co-IP	promote degradation	inhibit cholesterol esterification and storage	murine peritoneal macrophages cultured with or without ac-LDL for 18 h	mitigate AS	Akt3	[28]
ABCA1/G1	not applicable	co-IP	promote degradation	inhibit ABCA1/G1-mediated cholesterol efflux	CHO cells incubated with cholesterol/cyclodextrin for 8 h	aggravate AS	not applicable	[32]
ABCA1	not applicable	co-IP	promote degradation	inhibit ABCA1-mediated cholesterol efflux	HEK293 cells	aggravate AS	CSN	[35]
ABCA1	not applicable	co-IP	promote degradation	inhibit ABCA1-mediated cholesterol efflux	COS1 cells; liver plasma membrane fractions from mice	aggravate AS	LXRβ	[33]
ABCA1	not applicable	co-IP	promote degradation	inhibit ABCA1-mediated cholesterol efflux	J774 cells	aggravate AS	not applicable	[36]
ABCA1	not applicable	co-IP	promote degradation	inhibit ABCA1-mediated cholesterol efflux	mouse peritoneal macrophages	aggravate AS	not applicable	[34]
SR-B1	not applicable	co-IP	promote degradation	inhibit SR-B1-mediated cholesterol efflux	RAW264.7 cells; THP-1 macrophages treated with ox-LDL	aggravate AS	avoid magnetite NPs exposure	[37]
HIF1α	not applicable	not applicable	promote degradation	promote foam cell formation	RAW264.7 cells	aggravate AS	avoid BUVSs exposure	[38]
LXRα/β	not applicable	co-IP	promote degradation	inhibit ABCA1/G1-mediated cholesterol efflux	mouse peritoneal macrophages	aggravate AS	TRIM13 deficiency	[25]
PPARγ	not applicable	co-IP	promote degradation	inhibit ABCA1/G1-mediated cholesterol efflux	THP1 macrophages cultured with ox-LDL	aggravate AS	EOFAZ	[39]
PPARγ	Lys48, Lys63, polyUb	co-IP	not applicable	inhibit cholesterol efflux via downregulating ABCA1, ABCG1, LDLR, and PCSK9	RAW264.7 cells cultured with ox-LDL	aggravate AS	miR-30a-5p	[40]
cGAS	Lys48, polyUb	co-IP	promote degradation	inhibit inflammatory responses via inactivating TBK1-IRF3 pathway	RAW264.7 cells stimulated by ox-LDL	mitigate AS	ALDH2	[43]
STING	not applicable	co-IP	promote degradation	inhibit pro-inflammatory status	RAW264.7 cells	mitigate MI	SIRT6 deficiency	[44]
PPM1A	Lys63, polyUb	co-IP	promote stability	inhibit type-I IFN-mediated antiviral immunity via dephosphorylating TBK1-IRF3 pathway	THP-1 macrophages	aggravate viral myocarditis	TRIM18 deficiency	[45]
IRF7	monoUb	co-IP	promote activation	promote infiltration, inflammatory responses, and profibrotic potential via upregulating CCL5 and IFN signaling	BMDMs from mice with LPS stimulation	aggravate NICM	WWP2 deficiency	[46]
IRF5	Lys63	co-IP	promote activation	promote M1 polarization and migratory ability	BMDMs from mice stimulated with conditioned medium for 24 h	aggravate MIRI	Peli1 deficiency	[47]
TRAF2	Lys63	co-IP	promote activation	promote inflammatory responses via activation of MAPK and NF-κB pathways	HEK293T cells	mitigate acute ischemic stroke	SNHG15 deficiency	[58]
TRAF2/6	Lys63, polyUb	co-IP	promote activation	promote macrophage inflammation via NF-κB activation	THP-1 macrophages treated with LPS	aggravate AS	circARCN1 deficiency	[59]
TRAF6	polyUb	co-IP	promote activation	promote inflammatory responses via NF-κB activation	BMDMs from mice stimulated with LPS; HEK293T cells	exacerbate AS	MVP	[60]
IκBα	not applicable	co-IP	promote degradation	activate NF-κB signaling, promoting pyroptosis, inflammation, and foam cell formation	THP-1 macrophages treated with ox-LDL	aggravate AS	TRIM64 deficiency	[56]
SRSF1	not applicable	co-IP	promote degradation	promote inflammatory responses via NF-κB activation	BMDMs	aggravate DCM/EAM	MAAMT deficiency	[57]
IFNGR1	Lys48, polyUb	co-IP	promote degradation	inhibit pro-inflammatory state	HEK293T cells	mitigate MI	RNF149	[65]
XRCC1	not applicable	Western blotting	promote degradation	promote necroptosis and pro-inflammatory status via PARP1 activation	BMDMs from mice stimulated with ox-LDL	aggravate AS	TRIM25 deficiency	[66]
C/EBP-β	not applicable	not applicable	not applicable	promote M2 polarization	not applicable	mitigate ICH	Nrdp1	[67]
NAMPT1	not applicable	co-IP	promote degradation	promote macrophage infiltration and pro-inflammatory status	HEK293T cells	aggravate AAA	CTRP13 stimulation	[69]
YAP	Lys63, polyUb	co-IP	increase nuclear translocation and stability	promote chemokine production and migration	mouse peritoneal macrophages; HEK293 cells	aggravate AS	YAP deficiency	[61]
NLRP3	Lys63, polyUb	co-IP	inhibit activation	inhibit inflammatory responses	293T cells; J774A.1 cells	mitigate AS	tranilast	[71]
Nrf2	not applicable	co-IP	promote degradation	increase oxidative stress	RAW264.7 cells	aggravate AS	oridonin	[75]
p53	polyUb	co-IP	promote degradation	suppress lipid-bearing macrophages apoptosis	mouse peripheral blood monocytes cultured with and ag-LDL	aggravate AS	LIG deficiency	[77]
LXRα	polyUb	co-IP	promote degradation	promote ER stress-dependent apoptosis	mouse peritoneal macrophages	aggravate neointimal hyperplasia	IFN-γ deficiency	[79]
Keap1	not applicable	co-IP	promote degradation	inhibit ferroptosis and ferroptosis-mediated foam cell formation and inflammation	mouse peritoneal macrophages	mitigate AS	PNS	[82]
Sirt1	ubiquitination	co-IP	promote degradation	inhibit autophagy	primary human macrophages incubated with ox-LDL	aggravate AS	USP22 upregulation via CTRP9	[85]
MFN1/2	polyUb	co-IP	not applicable	promote mitophagy	BMDMs incubated with ox-LDL	mitigate AS	AIBP	[87]
LRP-1	not applicable	co-IP	promote degradation	decrease efferocytosis	RAW264.7 cells incubated with ox-LDL	aggravate AS	Epsins deficiency	[89]
PPARγ	not applicable	not applicable	promote degradation	promote pro-inflammatory status and impair efferocytosis	not applicable	aggravate AS	SHP2 deficiency	[90]

Abbreviations: Co-IP, co-immunoprecipitation; AS, atherosclerosis; SR, scavenger receptor; USP9X, ubiquitin-specific peptidase 9 X-linked; CD36, cluster of differentiation 36; USP, ubiquitin-specific proteases; UCH, ubiquitin C-terminal hydrolases; NP, nanoparticle; ABC, ATP-binding cassette transporters; LXR, liver X receptor; CSN, COP9 signalosome; AGE, advanced glycation end product; Par1, protease-activated receptor 1; TRIM, tripartite motif-containing protein; PPARγ, peroxisome proliferator-activated receptor γ; EOFAZ, essential oil from Fructus Alpinia zerumbet; PTEN, phosphatase and tensin homolog; SOCS1/3, suppressor of cytokine signaling 1/3; HIF1α, hypoxia-inducible factor 1α; BUVS, benzotriazole ultraviolet stabilizer; ACAT-1, acyl coenzyme A: cholesterol acyltransferase-1; Nrf2, nuclear factor erythroid 2-related factor 2; Keap1, Kelch-like ECH-associated protein 1; Sirt1, Sirtuin 1; CTRP, C1q/TNF-related protein; LIG, LDL-inducible gene; MFN, mitofusin; LRP-1, LDLR (low-density lipoprotein receptor)-related protein 1; NF-κB, nuclear factor-κB; IκB, inhibitor of κB; PARP1, poly (ADP-ribose) polymerase 1; cGAS, cyclic GMP-AMP synthase; STING, stimulator of interferon genes; C/EBP-β, CCAAT enhancer-binding protein-β; Nrdp1, neuregulin receptor degradation protein 1; DCM, dilated cardiomyopathy; EAM, experimental autoimmune myocarditis; SRSF1, serine/arginine-rich splicing factor 1; TBK1, TANK-binding kinase 1; IRF3, interferon regulatory factor 3; PPM1A, protein phosphatase 1A; IFN, interferon; NAMPT1, nicotinamide phosphoribosyl-transferase 1; AAA, abdominal aortic aneurysm; SHP2, Src homology 2-containing protein tyrosine phosphatase 2; NICM, non-ischemic cardiomyopathy; IRF, interferon regulatory factor; MIRI, myocardial ischemic and reperfusion injury; IFNGR1, interferon gamma receptor 1; MI, myocardial infarction; RNF149, ring finger protein 149; YAP, Yes-associated protein; TRAF, tumor necrosis factor receptor-associated factor; SNHG15, small nucleolar RNA host gene 15; NLRP3, NOD-like receptor thermal protein domain-associated protein 3; MVP, major vault protein; co-IP, co-immunoprecipitation.

Despite these challenges, the intricate relationship between ubiquitination and macrophage-driven inflammation highlights the potential of ubiquitination-targeting therapies to address key pathophysiological processes in CVD. Advances in single-cell omics, spatial transcriptomics, and targeted drug design may provide the tools needed to overcome these obstacles. For example, single-cell transcriptomic approaches could help identify macrophage-specific ubiquitination signatures in different disease states, enabling the development of more precise therapeutic interventions. Similarly, spatial transcriptomics could provide insights into the localization of ubiquitination-related molecules within atherosclerotic plaques, guiding the design of targeted therapies that focus on specific regions of interest.

In addition, future research should focus on unraveling the molecular mechanisms underlying ubiquitination-mediated macrophage regulation. Identifying the key E3 ligases, DUBs, and ubiquitination linkages that are selectively expressed in macrophages or are critical for their function in CVD could pave the way for the development of highly specific modulators. Moreover, understanding the spatiotemporal dynamics of ubiquitination in macrophages—how these modifications change during disease progression and in response to therapeutic interventions—will be essential for optimizing treatment strategies.

Finally, the potential of ubiquitination-targeting therapies extends beyond CVD. These pathways are likely to play important roles in other macrophage-driven diseases, such as autoimmune disorders, cancer, and neurodegenerative diseases. By integrating insights from CVD research with findings in other fields, researchers could develop a more comprehensive understanding of ubiquitination biology and identify shared therapeutic opportunities across diverse diseases.

In conclusion, targeting ubiquitination pathways holds significant promise for the treatment of CVD and other macrophage-driven diseases. However, the challenges of specificity, off-target effects, and pathway crosstalk must be addressed to fully realize this potential. By integrating advanced technologies, deciphering complex molecular networks, and developing context-specific therapeutic strategies, targeting ubiquitination could revolutionize the treatment of CVD and beyond.

## Abbreviations

The following abbreviations are used in this manuscript:

CVD	Cardiovascular disease
Ub	Ubiquitin
DUB	Deubiquitinating enzyme
USP	Ub-specific protease
OTU	Ovarian tumor protease
UCH	Ub C-terminal hydrolase
MJD	Machado–Joseph domain-containing protease
JAMM	JAMM/MPN domain-associated Zn-dependent metalloprotease
MINDY	Motif interacting with Ub-containing novel DUB family
MCPIP	Monocyte chemotactic protein-induced protein
PPPDE	Permuted papain fold peptidases of dsRNA virus and eukaryote
ZUP1	Zinc finger-containing Ub peptidase 1
AS	Atherosclerosis
M-CSF	Macrophage colony-stimulating factor
LXRα/β	Liver X receptor α/β
Ox-LDL	Oxidize low-density lipoprotein
SR	Scavenger receptor
Lox-1	Lectin-like ox-LDL receptor 1
CD36	Cluster of differentiation 36
SOCS1/3	Suppressor of cytokine signaling 1/3
TRIM	Tripartite motif-containing protein
PTEN	Phosphatase and tensin homolog
Ac-LDL	Acetylated LDL
ACAT-1	Acyl coenzyme A: cholesterol acyltransferase-1
ABCA1/G1	ATP-binding cassette transporters A1/G1
CSN	COP9 signalosome
AGE	Advanced glycation end product
Par1	Protease-activated receptor 1
NP	Nanoparticle
HIF1α	Hypoxia-inducible factor 1α
BUVS	Benzotriazole ultraviolet stabilizer
EOFAZ	Essential oil from Fructus Alpinia zerumbet
cGAS	Cyclic GMP-AMP synthase
STING	Stimulator of interferon genes
TBK1	TANK-binding kinase 1
IRF3	Interferon regulatory factor 3
MI	Myocardial infarction
PPM1A	Protein phosphatase 1A
MAVS	Mitochondrial antiviral signaling
IFN	Interferon
IRF	Interferon regulatory factor
NICM	Non-ischemic cardiomyopathy
PAD	Peripheral artery disease
IL-1R	Interlukein-1 receptor
TRAF	Tumor necrosis factor receptor-associated factor
MAPK	Mitogen-activated protein kinase
NF-κB	Nuclear factor-κB
IκB	Inhibitor of κB
SNHG15	Small nucleolar RNA host gene 15
MVP	Major vault protein
SRSF1	Serine/arginine-rich splicing factor 1
EAM	Experimental autoimmune myocarditis
IFNGR1	Interferon gamma receptor 1
JAK1	Janus kinase 1
STAT1	Signal transducer and activator of transcription 1
RNF149	Ring finger protein 149
Nrdp1	Neuregulin receptor degradation protein 1
C/EBP-β	CCAAT enhancer-binding protein-β
ICH	Intracerebral hemorrhage
NAMPT1	Nicotinamide phosphoribosyl-transferase 1
AAA	Abdominal aortic aneurysm
YAP	Yes-associated protein
NLRP3	NOD-like receptor thermal protein domain-associated protein 3
Nrf2	Nuclear factor erythroid 2-related factor 2
Keap1	Kelch-like ECH-associated protein 1
PARP1	Poly (ADP-ribose) polymerase 1
RIPK3	Receptor-interacting protein kinase 3
Ag-LDL	Aggregated LDL
LIG	LDL-inducible gene
PCI	Percutaneous coronary intervention
PNS	Panax notoginseng saponins
Sirt1	Sirtuin 1
CTRP	C1q/TNF-related protein
AIBP	Apolipoprotein A-I binding protein
PARK2	Parkin 2
MFN1/2	Mitofusin 1/2
ROS	Reactive oxygen species
LRP-1	LDLR (low-density lipoprotein receptor)-related protein 1
PPARγ	peroxisome proliferator-activated receptor γ
SHP2	Src homology 2-containing protein tyrosine phosphatase 2
MIRI	Myocardial ischemic and reperfusion injury
DCM	Dilated cardiomyopathy
EAM	Experimental autoimmune myocarditis
